# Strike Now for a Medical Bill

**Published:** 1897-02

**Authors:** 


### EDITORIAL DEPARTMENT.

F. E. DANIEL, M. D., Editor.
S. E. HUDSON, M. D., Managing Editor.
A. J. SMITH. M. D., Galveston, Associate Editor.

EDITORIAL STAFF:

PROF. J. E. THOMPSON, M. D., Texas Medical College, Galveston; Surgery.
PROF. WM. KEILLER, M. D., Texas Medical College, Galveston; Obstetrics and Gynecology.
PROF. DAVID CERNA, M. D., Texas Medical College, Galveston; Therapeutics.
PROF. A. J. SMITH, M. D., Texas Medical College, Galveston; Medicine.
DR. R. H. L. BIBB, City of Mexico; Foreign Correspondent.

Published Monthly at Austin, Texas, by Drs. Daniel and Hudson. Subscription price $2.00 a year in advance.

Eastern Agents: The Monihan-Fairchild Special Agency, 24 Park Place, New York City.

Official organ of the West Texas Medical Association, the Houston District Medical Association, the Austin District Medical Society, Brazos Valley Medical Association, the Galveston County Medical Society, and several others.

### EDITORIAL.

STRIKE NOW FOR A MEDICAL BILL.—The State Medical Association's bill to regulate the practice of medicine, adopted at the meeting at Fort Worth, in April last, was introduced in the Senate on the 24th of January, ult., by Senator Woods, of Grayson county, and was referred to the Senate Committee on Public Health. The bill has been printed, but at this writing no action has been taken on it by the committee.

Dr. J. S. Morris, of Cass county, is senator from his district, and is chairman of the Committee on Public Health; Dr. W. D. Yett is senator from Williamson and Travis counties, and is a member of the committee. In the House of Representatives, there are five physicians, Drs. Field, of Grayson county; Hill, of Travis county; Flint, of Marion county; D. A. C. Oliver, of Cass county, and Dr. Sherburne, of Denton county. All of these gentlemen are on the House Committee on Public Health, and when the bill gets into the House, of course it will go through their hands. *Now is the time to strike.* Let every reader of this in Texas, who feels an interest in the subject, and every reputable physician should have pride enough in his profession to take a deep interest in it, write, at once, a strong letter to his senator and representative, urging them to support the measure, and point out to them, at the same time, the urgent necessity for a good law on the subject, so as to protect the people from the ravages of the countless, conscienceless quacks and pretenders who are fleecing the people of money, and killing the confiding by their want of medical knowledge. The quack is one of the greatest dangers to the public health. The present law is inoperative and worthless; it recognizes *any kind* of a diploma as authority to practice! This is an outrage that no self-respecting citizen of Texas, and certainly no law-maker, should be willing to submit to. A diploma, even from the best college, conveys no license to practice. A diploma is only "evidence of a degree having been conferred," and every State should reserve the privilege of granting license to practice medicine. Why should Texas accept as a license to practice a certificate that the State of ———, through a chartered medical college, has conferred a degree on the bearer? It is an outrage.

Write at once, and urge your representative and senator to help the medical profession run out the quacks; pass a law that will not permit any man to trifle with the health and life of the people; a law that will limit the privilege of practicing medicine to persons who are qualified for the grave responsibilities of a physician, and can give proof of that qualification to a State authority.

And in writing, state to your representative and senator whether or not you are opposed to having a mixed board. If the majority of the regular physicians of the State desire a board composed of representatives of the so-called "three schools,"—why, the minority must, in duty, acquiesce. But, let it be remembered, in writing, that the homeopaths have "declined the honor;" they have *rejected* with scorn and indignation the overtures made to them, and the proposition to give them four members on a board of examiners to six regular physicians; they want and ought to have a separate board. There seems to be a disposition to accept whatever the legislature will give. We do not subscribe to this. Let us at least *ask for* what we want. Put yourself on record on this point. Let your spokesmen in the legislature know your preferences, at least; and if you are opposed to homeopaths being recognized in the way it is proposed in the bill, say so; but insist on their voting for a good medical bill of some sort.

The Journal takes occasion to compliment Dr. Cole on his very excellent off-hand clinical lecture on the management of a normal labor. It is eminently sensible, and we commend it, especially, to our younger readers. They have heard so much obstetrics that they are apt to lose sight of the fact that parturition is, for the most part—a physiological, and not a pathological process. Some of our older readers may read it with profit, also. Come again, Doctor.

---

Dr. Jackson, of Houston, treats the Journal's readers this month to a most excellent disquisition on rheumatism, a troublesome disease to manage. His treatment of the subject is lucid, rational and exhaustive. It does him credit. It is a most instructive paper. We were struck, however, by one remark—which seemed a little inconsistent. The doctor, while cautioning the nurse to handle the patient tenderly, so as to spare him all pain, advises the use of compound cathartic pills—a griping abomination—the effect of which—compared to rheumatism, would be like dynamite to a poppin' cracker. Ugh! C. C. P., U. S. P. *Nit.*

